# Immune responses following DNA vaccination by needle-free injection against *Burkholderia pseudomallei* hemolysin co-regulated protein 1

**DOI:** 10.3389/fimmu.2025.1612540

**Published:** 2025-06-25

**Authors:** Michael L. Davies, Sergei S. Biryukov, Christopher P. Klimko, Jennifer L. Dankmeyer, Nathaniel O. Rill, Melissa Hunter, Christopher T. Braun, Deven W. Patrick, Tyrique D. David, Steven A. Kwilas, Carlos I. Rodriguez, Brian A. Smith, Ju Qiu, Jay W. Hooper, Christopher K. Cote

**Affiliations:** ^1^ Bacteriology Division, United States Army Medical Research Institute of Infectious Diseases (USAMRIID), Fort Detrick, Frederick, MD, United States; ^2^ Virology Division, United States Army Medical Research Institute of Infectious Diseases (USAMRIID), Fort Detrick, Frederick, MD, United States; ^3^ Biostatistics Division, United States Army Medical Research Institute of Infectious Diseases (USAMRIID), Fort Detrick, Frederick, MD, United States

**Keywords:** melioidosis, *Burkholderia pseudomallei*, vaccines, DNA vaccines, needle-free injection, mice, immunity

## Abstract

*Burkholderia pseudomallei* is a facultative intracellular bacterium found in soil, which causes melioidosis, a disease with diverse symptomatology. *B. pseudomallei* is an emerging threat in the United States based on recent environmental samples and case reports. Acute infection is 10%–40% fatal depending on treatment conditions. No vaccines for *B. pseudomallei* have been approved for human use, although several are under development, mostly targeting the antigens Hcp1 (hemolysin-coregulated protein 1) and CPS (capsular polysaccharide). For development of new vaccines, DNA compares favorably to other platforms in storage stability, low cost, and ease of design. Needle-free jet injection has been effective in immunizing against several infections in laboratory animals; the delivery devices are simple to use and have been FDA 510k cleared for human use. Herein, we developed a DNA vaccine targeting Hcp1 (pWRG/Hcp1) and delivered it to rabbits and mice by jet injection using a PharmaJet Stratis and a prototype adjustable-dose PharmaJet Tropis, respectively. The Hcp1 DNA vaccine was unadjuvanted and not combined with any other *B. pseudomallei* antigens. Immunization was followed by assessment of serum antibodies and cellular immunity against Hcp1 protein. Rabbits and mice showed induction of anti-Hcp1 antibodies after as few as two doses of pWRG/Hcp1, and splenocytes responsive to restimulation with Hcp1 protein were also detected after two doses. These results demonstrate the feasibility of inducing immunity against Hcp1 of *B. pseudomallei* using DNA alone. These results also serve as a proof-of-concept for immunizing mice with a PharmaJet device previously only used for larger animals.

## Introduction

*Burkholderia pseudomallei* is a motile facultative intracellular gram-negative bacterium found in various environmental niches including tropical soils. *B. pseudomallei* is the etiological agent of melioidosis and can infect humans and many animal species (1, 2). *B. pseudomallei* has been historically endemic in southeast Asia and northern Australia, but has recently been identified in other tropical and subtropical parts of the world including Africa and the Americas (3). In context of recent events, melioidosis can be considered an emerging threat in the United States (4–6). Infection occurs following environmental exposure via ingestion, inhalation, or percutaneous inoculation. Clinical manifestations of melioidosis range from acute and rapidly fatal, with septicemia and pneumonia, to protracted chronic or latent forms, with recrudescence at various times post-infection (1). Symptoms are diverse and nonspecific and are influenced by the route of infection as well as the presence of comorbidities such as diabetes, alcoholism, or kidney disfunction (2). *B. pseudomallei* accounts for approximately 89,000 deaths and 165,000 cases a year, but these numbers are thought to be dramatically underreported (3, 7). Under ideal treatment conditions acute infection mortality is roughly 10% and increases to 40% in regions where diagnostics and antibiotics are scarce (7, 8).

Patient care of *B. pseudomallei* infection can be difficult and often requires a prolonged antibiotic regimen. The revised 2020 Darwin Guidelines recommend an initial intensive phase with intravenous antibiotics for a minimum of 2 weeks, followed by an eradication phase with oral antibiotics for a minimum of 3 months, both timelines being dependent on severity of the disease (2, 9). Although antimicrobial resistance to clinically significant antibiotics is currently rare, the *B. pseudomallei* K96243 genome encodes putative genes capable of several resistance mechanisms (10, 11). This potential for acquired resistance may be coming to fruition given that there are emerging reports of resistance due to increased use of ceftazidime to treat *B. pseudomallei* (11–17). Furthermore, there are no vaccines currently approved for human use against *B. pseudomallei*.

In the past, most vaccine development efforts have focused on inactivated whole-cell, live attenuated, subunit or glycoconjugate vaccines (18). Several candidate *B. pseudomallei* vaccines are at various stages of development with some transitioning to phase 1 clinical trials, such as the protein and glycoconjugate subunit vaccine formulation delivered subcutaneously (2). This vaccine employs two essential *B. pseudomallei* virulence factors as follows: hemolysin-coregulated protein 1 (Hcp1), a component of the Type 6 secretion system, and capsular polysaccharide (CPS), with CPS conjugated to carrier protein CRM197, a nontoxic variant of diphtheria toxin. Hcp1 and CPS–CRM197 are admixed with adjuvants CpG (TLR9 agonist) and Alhydrogel (an aluminum hydroxide wet gel suspension) (19). Antibodies directed against Hcp1 and CPS are present in melioidosis convalescent patient sera (20, 21). Although these vaccines confer varying levels of protection, sterile immunity remains elusive in laboratory animals (19, 22, 23). Novel vaccine formulations and vaccine delivery methods are needed to enhance protective efficacy.

DNA vaccines have several advantages over other vaccine platforms including storage stability, low cost, ease of design, and extended immune response (18, 24, 25). Historically, one of the greatest challenges for nucleotide-based vaccines has been the delivery method. Several methods have since been developed of which perhaps the most successful is the lipid nanoparticle delivery system used in the COVID-19 vaccines mRNA-1273 and BNT162b (26–28). These widely distributed and successful vaccines demonstrate the utility and efficacy of gene-based vaccines. The utility of a DNA vaccine platform against *B. pseudomallei* has been less extensively researched and predominately targeted *B. pseudomallei* flagellin protein FliC delivered via intramuscular or intranasal routes (29, 30). Hcp1 is another obvious candidate protein for targeting with DNA vaccination and could be combined with stable well-established vaccine technology targeting bacterial polysaccharides.

Here, we present proof-of-concept work demonstrating immunogenicity of a DNA vaccine against the *B. pseudomallei* Hcp1 antigen in mice. In this study, we evaluated the immune response in rabbits and mice vaccinated via the PharmaJet Stratis or Tropis devices, respectively. Both are disposable syringe jet injection devices also known as needle-free injection systems (NFIS). The vaccine was an unadjuvanted plasmid DNA construct containing the *B. pseudomallei* gene *hcp1*. The rationale for using jet injection is that we and others have found that DNA vaccines delivered by jet injection (DNA/jet) are more effective than needle and syringe and are more pragmatic than other modes of delivery such as particle-mediated epidermal delivery or electroporation (31, 32). Jet injection involves the delivery of a high-velocity liquid jet into the tissue. It is likely that the rapid influx of liquid results in an increased amount of nucleic acid physically delivered intracellularly (33). The Pharmajet Stratis is designed to deliver 0.5 ml of vaccine intramuscularly (or subcutaneously for some indications). The smallest animal on which we have used this device is a rabbit where we have delivered numerous vaccines intramuscularly (31, 34). The PharmaJet Tropis is also FDA 510k cleared for human use. It is designed to deliver 0.1 ml of volume intradermally to humans. We have used this intradermal device to successfully deliver 0.1 ml of hantavirus vaccine intramuscularly/subcutaneously to Syrian hamsters, which are approximately 100 g (35). In this study, for the first time in mice, we employed a prototype adjustable-dose Tropis device provided by PharmaJet that can deliver a range of volumes. We used this adjustable Tropis device to deliver 0.05 ml of vaccine intramuscularly/subcutaneously to mice, which are approximately 20 g.

## Materials and methods

### DNA vaccine plasmid

The *hcp1* gene used in this study (PubMed PMID: QRM26608) had its open reading frame codon optimized for *Homo sapiens*. Gene optimization and synthesis were performed using a contract service (TWIST). The *hcp1* gene was inserted between the *Not*I and *Bgl*II sites of the DNA vaccine vector pWRG7077 to create the DNA vaccine pWRG/Hcp1.

### Immunofluorescent antibody test

Construct expression was confirmed by transfecting 293T cell monolayers with pWRG/Hcp1 using FuGene 6 (Promega, Madison, WI). The transfection was performed as described in the package insert. Briefly, 293T cells were split and plated at ~100,000 cells per well of a 96-well plate. After an overnight incubation at 37°C, the cells were ~70% confluent. In a round-bottom 96-well plate, the transfection complexes were made with a starting DNA concentration of 20 ng/μl. A volume of 10 µl with 200 ng was added to the medium across the 96-well plate. Certain wells were excluded like the cell-only control and the wells receiving the non-specific DNA plasmid pWRG/EBOV. After an overnight incubation at 37°C, the cells were fixed with 10% formalin and immunostained using a purified mouse anti-Hcp1 antibody (Brett and Burtnick, University of Nevada School of Medicine, Reno) and goat anti-mouse Alexa 488 conjugate (Invitrogen, Carlsbad, CA) as the secondary antibody. All samples were tested in duplicate.

### Animals

Two female New Zealand white rabbits, ~11 weeks of age (Charles River, Frederick, MD), were vaccinated with pWRG/Hcp1 (1 mg/0.5 ml dose per DNA vaccination in PBS) using the PharmaJet Stratis jet injection device. Rabbits were vaccinated at 4-week intervals in the lateral thigh muscles. The opposite thigh was used from the previous vaccination during this vaccination series. Sera were collected at timepoints after each vaccination and evaluated for anti-Hcp1 antibody titers.

BALB/c mice, approximately 7–9 weeks of age at first vaccination, were purchased from Charles River. The mice received 100 μg of DNA in PBS delivered as a 50-μl intramuscular/subcutaneous (IM/SC) dose by an adjustable-dose PharmaJet Tropis in the thigh muscle of the rear flank. The injection site had been shaved prior to vaccination with electric fur clippers. Mice were vaccinated at 3-week intervals. Sera were collected at timepoints after each vaccination and evaluated for anti-Hcp1 antibody titers. Splenocytes were evaluated at the same timepoints and restimulated *ex vivo* to evaluate cellular immunity.

### Humoral immunity assays

Antibody levels in sera were measured by semiquantitative ELISA as described previously (36). In brief, 96-well plates were coated with a 2µg/ml solution of recombinant Hcp1 protein, and serial 1:2 dilutions of sera were applied to the plate in triplicate, followed by detection with secondary goat anti-mouse or anti-rabbit IgG (Southern Biotech, Birmingham AL). Recombinant Hcp1 was purified from *Escherichia coli* as previously described (21, 37) was a kind gift from Paul Brett and Mary Burtnick (University of Nevada School of Medicine, Reno). Antibody titer results were reported as the reciprocal of the highest dilution resulting in a mean OD of at least 0.100 at 450 nm (delta of OD at 450 nm minus OD of reference wavelength of 570 nm).

### Cellular immunity assays

Splenocytes from immunized mice were isolated and resuspended for analysis by ELISpot and Luminex as described previously (38). For restimulation, cells were incubated in the presence of Hcp1 protein (see above) or gamma radiation-inactivated *B. pseudomallei* K96243 cells (irBpK); the medium alone was negative control, and the positive control for cells having the capacity for restimulation was a solution of PMA (100 ng/ml) and ionomycin (0.5 µg/ml).

For ELISpot quantification of T cells secreting IFN-γ, splenocytes were incubated for 1 day in CTL-Test medium (CTL, Shaker Heights, OH) with 1% L-glutamine and 10 µg/ml of Hcp1 protein, followed by incubation with anti-mouse IFN-γ detection antibody and colorimetric detection, as described previously (39). Spots were scanned and counted using an automated ELISpot reader (ImmunoSpot S6, CTL), normalized, and reported as spot-forming cells (SFC) per 10^6^ total splenocytes.

For multiplex quantification of cytokines secreted by splenocytes upon restimulation, splenocytes were incubated in RPMI-1640 complete medium with 10% fetal bovine serum (39) in the presence of 10 µg/ml of Hcp1 protein or irBpK cells (see above). After 2 days of restimulation, supernatants were isolated and purified by centrifugation, then assessed for secreted levels of cytokines and chemokines (ENA-78/CXCL5, Eotaxin, G-CSF, GM-CSF, GRO-α/CXCL1, IFN-α, IFN-γ, IL-1α, IL-1β, IL-10, IL-12p70, IL-13, IL-15, IL-17A, IL-18, IL-2, IL-22, IL-23, IL-27, IL-28, IL-3, IL-31, IL-4, IL-5, IL-6, IL-9, IP-10/CXCL10, LIF, M-CSF, MCP-1/CCL2, MCP-3/CCL7, MIP-1α/CCL3, MIP-1β/CCL4, MIP-2α/CXCL2, RANTES/CCL5, TNF-α) using the ProcartaPlex Mouse Cytokine & Chemokine 36-Plex panel (Thermo Fisher, Waltham, MA). Data were collected using a MagPix instrument (Thermo Fisher) and analyzed in xPONENT software version 4.3. A five-parameter logistic regression model was used to generate a calibration curve for each analyte based on serial dilutions of a reference standard. Cytokine values above the upper limit of quantitation (ULOQ) were recorded as the ULOQ, and values below the lower limit of quantitation (LLOQ) were recorded as the LLOQ.

### Statistical analyses

ELISpot and Luminex data were log10 transformed prior to analysis. For ELISA, pairwise treatment groups were compared by negative binomial generalized linear mixed model to account for both overdispersion and random individual subject effects. Degrees of freedom were estimated using the Kenward–Roger method to improve accuracy in inference. For ELISpot and Luminex cytokine results, pairwise treatment groups were compared by linear mixed-effects model. No multiplicity adjustment was applied. Analysis was implemented using PROC GLIMMIX in SAS version 9.4 (SAS Institute Inc., Cary, NC).

## Results

### Hcp1 DNA vaccine construction and expression

**Figure 1:**
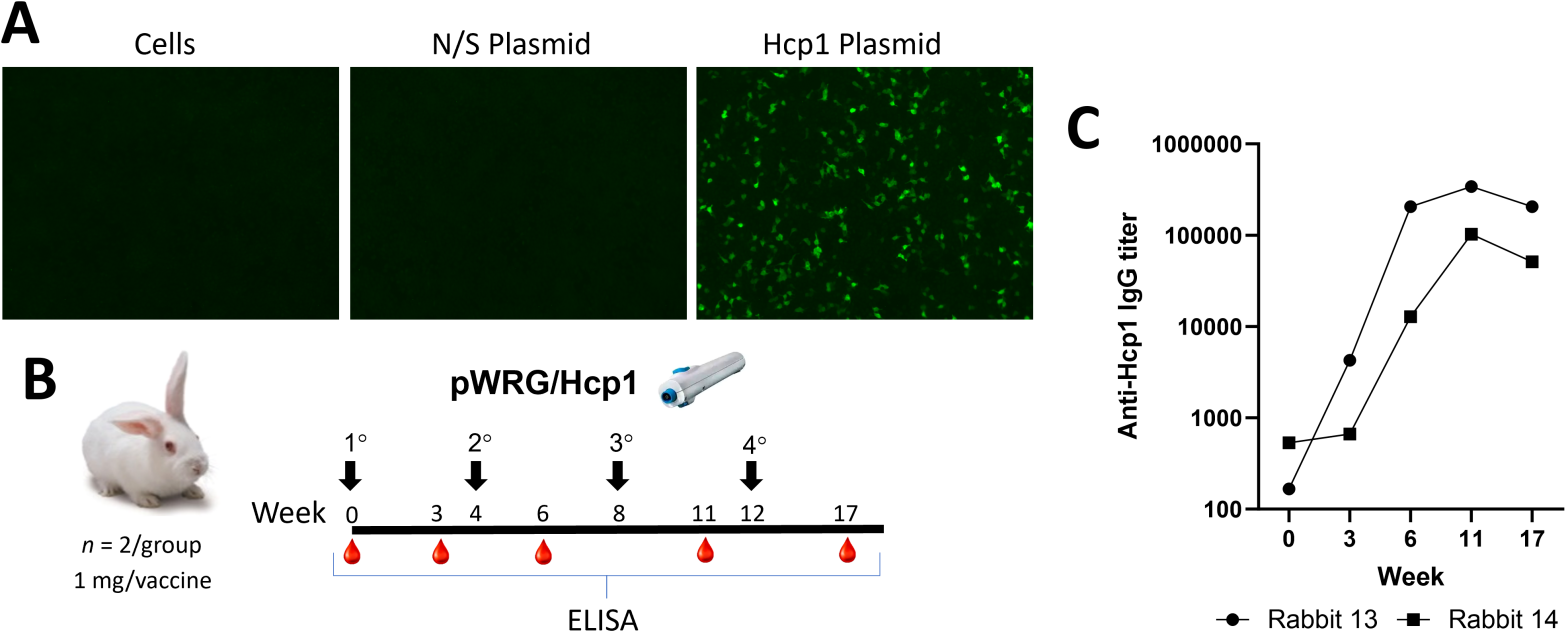
pWRG/Hcp1 DNA *in vitro* expression and immunogenicity testing in rabbits. **(A)** 293T cells were transfected with pWRG/Hcp1 or non-specific plasmid pWRG/EBOV and, after 24 h, stained with purified Hcp1 mouse antibody. Expression was only detected in the Hcp1 transfected cells, with no background staining observed. **(B)** Experimental design. Female New Zealand white rabbits were vaccinated with pWRG/Hcp1 (1 mg in 0.5 ml dose per DNA vaccination) at the indicated timepoint. Sera were collected and evaluated for anti-Hcp1 antibody by ELISA in naïve animals and after one, two, three, or four vaccinations. **(C)** Sera from vaccinated rabbits were measured using a semiquantitative ELISA with 2 µg/ml of Hcp1 as coating antigen. Results are shown as the reciprocal of the highest dilution giving an OD of at least 0.1 at 450 nm [delta of (OD_450_ − OD_570_)]. All samples were measured in triplicate.

The Hcp1 gene from PubMed PMID: QRM26608 was codon optimized and synthesized (Twist Bioscience, Quincy, MA) and then cloned into the DNA vaccine plasmid pWRG7077 vector to yield pWRG/Hcp1. Expression was confirmed by IFAT after transfecting pWRG/Hcp1 into 293T cells and staining with anti-Hcp1 mouse immune sera (**Figure 1A**).

### pWRG/Hcp1 DNA vaccine immunogenicity testing in rabbits

To determine if the pWRG/Hcp1 DNA vaccine was immunogenic, and to produce anti-Hcp1 immune sera, two female New Zealand white rabbits (*Oryctolaus cuniculus*) were vaccinated using the PharmaJet Stratis jet injection device (**Figure 1B**). The rabbits were vaccinated four times at 4-week intervals; sera were collected at timepoints after each vaccination and evaluated for anti-Hcp1 antibodies by semiquantitative endpoint ELISA. The rabbits responded after a single vaccination, and the anti-Hcp1 antibody titers increased by two orders of magnitude by the third vaccination and then plateaued. These data demonstrated that pWRG/Hcp1 was immunogenic when delivered by jet injection.

### The Hcp1 DNA vaccine construct results in detectable immune response in mice

**Figure 2:**
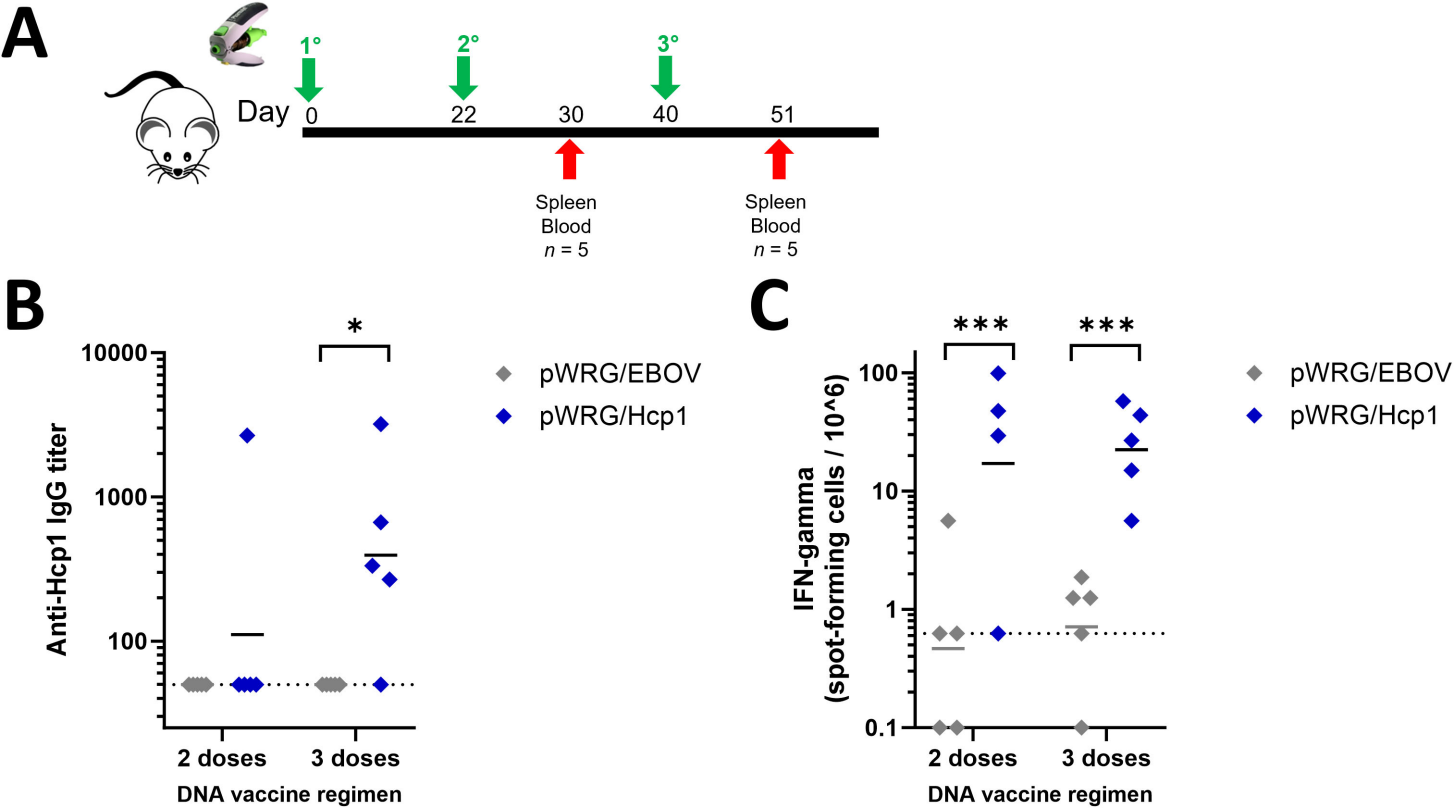
Mice immunized with pWRG/Hcp1 DNA have anti-Hcp1 serum antibodies and Hcp1-specific splenic T cells. **(A)** Experimental design. BALB/c mice received 100 μg of DNA delivered in 0.05 ml injections (IM/SC) to the thigh muscle of the rear flank. **(B)** Sera from mice immunized with pWRG/Hcp1 or pWRG/EBOV were measured using a semiquantitative ELISA with 2 µg/ml of Hcp1 as coating antigen as in **Figure 1**. All samples were measured in triplicate (*n* = 5). **(C)** Splenocytes from mice immunized with pWRG/Hcp1 or pWRG/EBOV were stimulated *ex vivo* with 10 µg/ml of soluble Hcp1 antigen for 24 h in plates coated with anti-mouse IFN-γ monoclonal antibody. Spot-forming cells were counted and normalized to spots/million cells. All samples were measured in duplicate (*n* = 4 or 5). Dotted lines indicate the limit of detection. *p < 0.05; **p < 0.001 in a pairwise comparison of groups by linear mixed-effects model.

To determine if the Hcp1 DNA vaccine was immunogenic in a standard animal model for *B. pseudomallei*, female BALB/c mice were vaccinated three times (prime, first boost, second boost) at 3-week intervals with the pWRG/Hcp1 DNA vaccine or negative control pWRG/EBOV using the adjustable Tropis jet injection device (**Figure 2A**). Sera and splenocytes were collected 8 days after each boost. Semiquantitative endpoint ELISA was used to assay for serum antibodies against Hcp1 after the second and third doses (**Figure 2B**). After two doses of vaccine, one of five pWRG/Hcp1 vaccinated mice had detectable anti-Hcp1 IgG. After the third dose, the number of mice responding to vaccination increased to four of five. All mice given irrelevant DNA (pWRG/EBOV) were negative in this assay. Importantly, the Hcp1 produced via the DNA/jet vaccine results in antibodies that can detect the Hcp1 protein produced in *E. coli*. These results show that the pWRG/Hcp1 DNA vaccine construct, delivered without additional adjuvants or protein antigens, can induce B-cell maturation and class switching.

To assess T-cell immunity, splenocytes from immunized mice were restimulated *ex vivo* with recombinant Hcp1 antigen, and the percent of splenocytes induced to produce IFN-γ during restimulation was quantified in each sample using ELISpot (**Figure 2C**). Mice given Hcp1 DNA, with either two or three doses of vaccine, had significantly more spots per million cells than mice vaccinated with the negative control plasmid.

**Figure 3:**
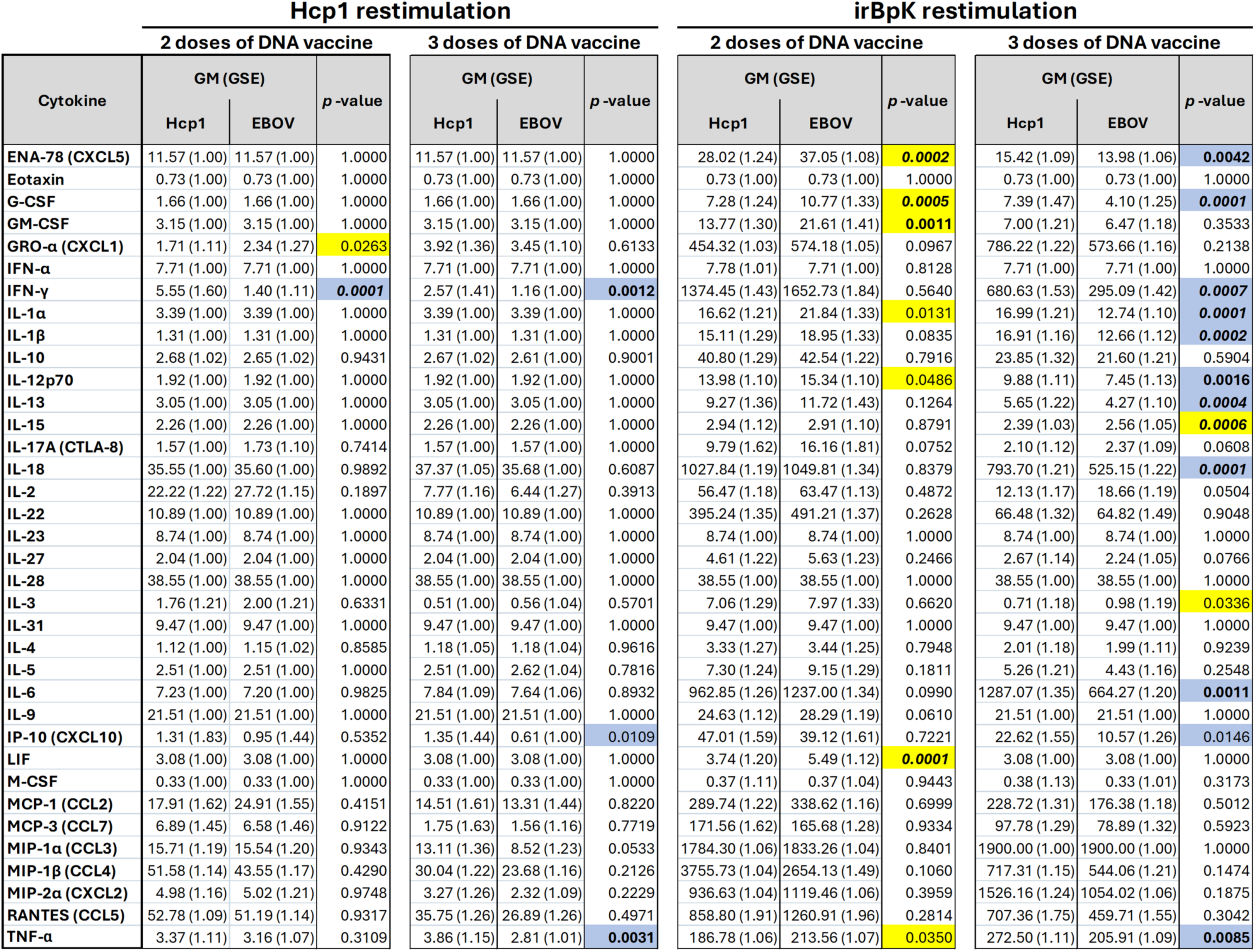
Cytokine levels secreted by splenocytes from mice vaccinated with pWRG/Hcp1 or pWRG/EBOV DNA vaccines. Mice were given two or three doses of pWRG/Hcp1 DNA or nonspecific control pWRG/EBOV DNA using the PharmaJet Tropis. Splenocytes from vaccinated mice were stimulated *ex vivo* by 10 µg/ml of soluble Hcp1 antigen or 5 µg/ml of irradiated *B. pseudomallei* K96243 cells. After 2 days, supernatants were isolated, and 36 cytokines were measured using a Luminex kit. The geometric mean (GM) and standard error (GSE) for each cytokine (measured as pg/ml of supernatant) are shown for each group, along with *p*-values for each comparison of Hcp1 to EBOV groups calculated using a linear mixed-effects model. Values above the ULOQ were replaced with the ULOQ, and values below the LLOQ were replaced with the LLOQ. All samples were measured in duplicate (*n* = 5). Blue: higher in Hcp1 group (*p*-value < 0.05). Yellow: higher in EBOV group (*p*-value < 0.05). Bold: *p*-value < 0.01. Bold/italics: *p*-value < 0.001.

In the context of melioidosis, several pro-inflammatory cytokines (e.g., IL-6, TNF-α, IL-1α, IL-1β, IL-17A) as well as anti-inflammatory (IL-10) and those indicating type 1 (IFN-γ) or type 2 immunity (IL-4, IL-5, IL-13) have been measured in patient survival studies (40–42) and in pre-clinical models assessing vaccination and treatment efficacy (43–45). Therefore, we investigated a wide range of representative cytokines and chemokines, including all of these, using a Luminex multiplex kit to quantify soluble factors produced by splenocytes after restimulation *ex vivo*. For this assay, splenocytes were restimulated with either recombinant Hcp1 or a preparation of whole *B. pseudomallei* K96243 cells that had been inactivated by radiation (“irBpK”). For each cytokine in the kit, geometric means were compared between mice immunized with either Hcp1 or EBV DNA (**Figure 3**).

**Figure 4:**
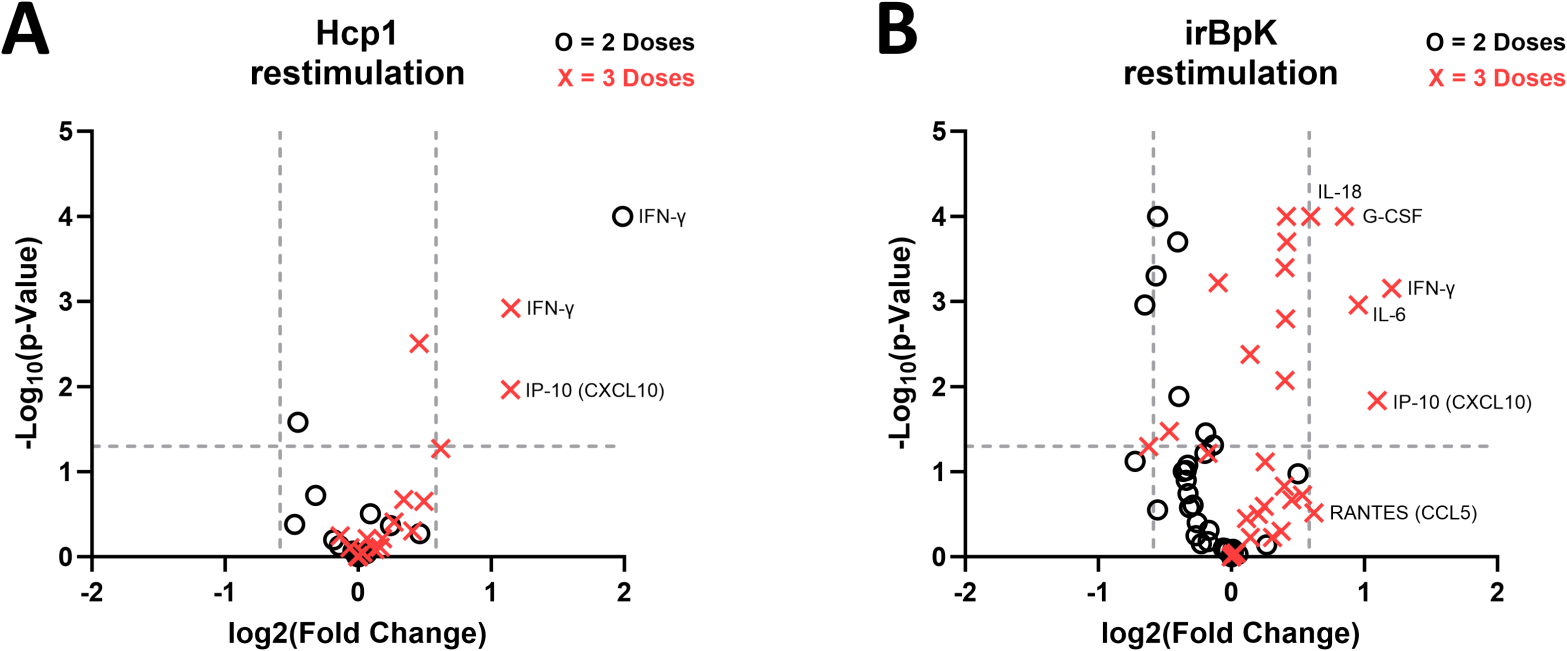
Fold change in *B. pseudomallei*-specific cytokine responses in splenocytes from mice given pWRG/Hcp1 DNA relative to mice given pWRG/EBOV. **(A)** Splenocytes from mice immunized with two doses (black circle) or three doses (red X) of pWRG/Hcp1 or pWRG/EBOV were stimulated *ex vivo* by 10 µg/ml of soluble Hcp1 antigen. After 2 days, supernatants were isolated, and 36 cytokines were measured using a Luminex kit. We measured the geometric mean (GM) for each cytokine, calculated the fold change for the pWRG/Hcp1 GM compared to the pWRG/EBOV GM, and generated a *p*-value for each comparison using a linear mixed-effects model. **(B)** Splenocytes from mice given two doses (black circle) or three doses (red X) of pWRG/Hcp1 or pWRG/EBOV were stimulated *ex vivo* by irradiated *B. pseudomallei* K96243 cells (5 µg/ml), and the above analysis was performed. Plots show significance on the y-axis, and fold change on the x-axis, of each comparison between pWRG/Hcp1 and pWRG/EBOV immunized splenocytes. All samples were measured in duplicate (*n* = 5). Dotted lines show *p* = 0.05 on the y-axis and fold change = 1.5 on the *x*-axis.

A volcano plot (**Figure 4A**) shows that for mice that received two doses of vaccine, there was limited difference between geometric means relative to the non-specific EBOV-vaccinated mice after Hcp1 restimulation. Splenocytes from the Hcp1 group produced significantly more IFN-γ, and splenocytes from the EBOV group produced more GRO-α, though the difference in fold change for GRO-α was minimal. After three doses of the vaccine, the only cytokines significantly different between groups were IFN-γ, TNF-α, and IP-10, all produced at higher levels by splenocytes in the Hcp1 group. These results support the ELISpot data to show a population of Hcp1-specific splenocytes present after peripheral vaccination with Hcp1 DNA but not EBOV DNA. IFN-γ is predominantly produced by activated T cells; TNF-α is produced by activated T cells and also IFN-γ-stimulated myeloid cells; and IP-10 is produced by a wide range of cells generally after IFN-γ stimulus (46–48).

Finally, splenocytes from both groups were restimulated with irBpK, which gave greater nonspecific activation, likely due to additional antigens and immunostimulatory factors such as LPS and flagellin found in the bacterial preparation. We employed this stimulus, although the higher background makes it harder to detect small differences between groups, because it reflects Hcp1 in its native rather than recombinant state. After two doses of vaccine, restimulated splenocytes from the Hcp1 group did not show any enhanced cytokine secretion compared to those of the negative control group (**Figure 4B**); the only differences were in the opposite direction and with minimal fold change. However, after three doses of vaccine, several cytokines (including IFN-γ, TNF-α, and IP-10 again, in addition to IL-6, IL-1α, IL-1β, IL-18, GM-CSF) were secreted at significantly higher levels by splenocytes from Hcp1-vaccinated mice.

## Discussion

The rapid onset of disease, severity of disease, extensive treatment regimen, expanding global distribution, potential for increased antibiotic resistance, and lack of a vaccine are all factors that make *B. pseudomallei* a global public health threat as well as a biodefense concern (classified as a Tier 1 Select Agent by the US Department of Health and Human Services). All these aspects underline the importance for effective and robust treatment and preventative methods against *B. pseudomallei*. A cost-effective and efficacious vaccine strategy would be an important development in both the public health and biodefense research communities.

DNA vaccines have a long history with a wide range of delivery technologies including needle and syringe, particle-mediated epidermal jet injection (gene gun), electroporation, microneedle patches, and jet injection in various configurations (32). For all these methods, there is an expected tradeoff between simplicity and cost versus the potency of the immune response. For example, a needle and syringe are simple and inexpensive, but the efficiency of intracellular DNA delivery is low, and the immunogenicity is poor. In contrast, electroporation is one of the most potent methods of delivering DNA vaccines; however, it is complex and relatively expensive. An advantage of the PharmaJet devices for DNA vaccines (DNA/jet) is that they are relatively simple, inexpensive, and elicit a significantly more potent immune response than needles and syringes. We have transitioned several virus-targeted DNA/jet vaccines from preclinical testing into the clinic (49, 50). A disadvantage of these vaccines is that boosts are required to achieve adequate immunity (e.g., neutralizing antibody responses); however, their safety and logistic profile are excellent.

Another drawback of DNA/jet vaccines is the difficulty in scaling down to small rodents during preclinical testing. This makes it more difficult to make comparisons with other vaccine platforms in small rodents such as mice. Here, we demonstrate that a modified intradermal jet injection device can be used to successfully deliver a DNA vaccine to mice providing another tool for vaccine research and development. This is supported by the data herein showing that mice immunized with pWRG/Hcp1 DNA, in the absence of any protein antigens or adjuvants, developed class-switched IgG antibodies against Hcp1 protein (**Figure 2B**), functional splenic T cells capable of responding to Hcp1 protein restimulation (**Figure 2C**), and splenocytes poised to activate the immune response with a variety of cytokines upon exposure to *B. pseudomallei* (**Figures 3**, **4**). A third dose of vaccine was seen to enhance both anti-Hcp1 IgG titers and Hcp1-specific splenocytes in this initial mouse study. However, rabbit ELISA data (**Figure 1**) suggest that a fourth dose of vaccine would be superfluous in further enhancing titers.

After restimulation of splenocytes with recombinant Hcp1, cells from Hcp1-vaccinated mice (compared to EBOV-vaccinated mice) had greater production of proinflammatory cytokine TNF-α and type 1 inflammatory cytokines IFN-γ and IP-10 (**Figures 3**, **4A**). After restimulation with irBpK, which includes native Hcp1 protein and also additional nonspecific immunostimulatory components, cells from Hcp1-vaccinated mice had greater production of several other cytokines (**Figures 3**, **4B**) that were all aligned with a type 1 inflammatory response with the exception of IL-13, a type 2 cytokine that has also been correlated with *B. pseudomallei* bacterial burden *in vivo* (43). In a recent study, we saw that several of these (IFN-γ, TNF-α, IP-10, IL-1β, IL-6, IL-18, IL-13) were also significantly induced in splenocytes from mice vaccinated with the current “gold standard” vaccine, Hcp1 protein combined with CPS-CRM197, Alhydrogel, and CpG given subcutaneously (23). Splenocytes induced by that vaccine also produced chemokines CCL7, CCL2, CCL4, CXCL2, and CCL3 as well as IL-2, IL-3, IL-4, and IL-22, which were not seen here, understandably given the greater immunogenic potential of that vaccine.

Anti-Hcp1 IgG titers induced by Hcp1 DNA vaccination in mice had a geometric mean of ~400 in the reciprocal limiting dilution assay, which was two logs lower than the anti-Hcp1 titers we have observed in mice given the “gold standard” vaccine (23). However, in a recent study using Hcp1 protein with staphylococcal membrane vesicles as a vaccine platform, the vaccine was 60% protective with anti-Hcp1 serum IgG titer <1,000, while the same vaccine with additional Freund’s adjuvant induced similar protection (70%) despite titers two logs higher (51). This suggests that a moderate amount of anti-Hcp1 serum antibody is sufficient when combined with cellular and trained immunity.

In this brief communication, we have demonstrated that a modified Pharmajet Tropis device can be used in mice and that the vaccination provided by the jet injection strategy results in both humoral and cellular immunity. Because of the complex bacterial pathogenesis of *B. pseudomallei* and clinical presentations of melioidosis, this sole vaccine antigen is not expected to offer significant protection to mice, as the current gold standard vaccine strategy involves not only Hcp1 but also capsular polysaccharide and adjuvants as described above. Future studies will focus on incorporating DNA vaccine constructs into the multi-component melioidosis vaccine and other vaccines being designed against other bacterial pathogens.

## Data Availability

The raw data supporting the conclusions of this article will be made available by the authors, without undue reservation.
